# PERFORMANCE OF FUNCTION-FOCUSED CARE BY NURSING STAFF

**DOI:** 10.1093/geroni/igad104.1108

**Published:** 2023-12-21

**Authors:** Elizabeth Galik

**Affiliations:** University of Maryland School of Nursing, Baltimore, Maryland, United States

## Abstract

To help acute care nurses learn to provide function focused care during care interactions the Function Focused Care intervention is implemented on nursing units. The Function Focused Care intervention is based on the Social Ecological Model, Social Cognitive Theory and the Evidence Integration Triangle and includes four components: (1) Development of a stakeholder team; (2) Education; (3) Development of functional care plans; and (4) Mentoring and motivating. Our nurse interventionist provides function focused care education as per the preference of the setting. Following education, direct observation during care interactions is done and the nurse interventionist provides recommendations related to the approach being used or provides positive reinforcement if function focused care is provided. The observation tool used to evaluate function focused care by the nursing staff includes 19 different care interactions (e.g., bathing, dressing, transferring). Guidelines are provided for what is and is not function focused care. In the first four treatment sites, at 2 months post implementation the nurses provided function focused care during 81% of the of care interactions observed, by 6 months this increased to 85% and by 10 months this increased to 88%. There was also a decline in the number of care interactions in which function focused care was not provided. The findings suggest that nurses can provide function focused care even during times of significant shortages and that continued oversight and education can help improve the use of this approach.

